# EHMTI-0391. Use of telemedicine in the management of headache: a new tool between general practitioners and neurologists

**DOI:** 10.1186/1129-2377-15-S1-J9

**Published:** 2014-09-18

**Authors:** E Murillo Espejo, MC González Oria, MM Sánchez Calle, A Martínez García, F Núñez Benjumea, D Jiménez Hernández

**Affiliations:** 1 Neurology, H.U. Virgen del Rocío, Sevilla, Spain

## Introduction

Headache is the most common neurological disorder seen by general practitioners (GPs) and neurologists. We propose the use of telemedicine system to evaluate patients with headache. This system will provide direct contact among GPs, Neurologists and patients, avoiding referal to the speciality clinic.

## Aims

Evaluation of telemedicine system in the assessment of patients suffering from headache, considering technical and assistance quality of care, delay in providing care, efficacy and patient’s satisfaction.

## Methods

We evaluated 15 patients with headache (13 women and 2 men, average age 36 years) by telemedicine system. Patients first evaluation was done by GPs and afterwards the same patients were evaluated by Neurologists using this system.

## Results

Migraine was diagnosed in 86.7% of patients, and tensional headache in 13.3%. In 53,3% of patients GPs and Neurologists made the same diagnosis. Neuroimaging studies were obtained in 3 patients, and the results were given to the patients using this system. The waiting time to be evaluated by Neurologists was 11 days. 80% of patients felt comfortable using this system, and 47% thougth it was better than face-to-face consultation. 66.7% of patients thought they could save time using this system and 100% will highly recommend it.

## Conclusions

Our preliminary results showed that telemedicine system could be an useful and effective tool in the management of patients with headache between GPs and Neurologists. It will reduce the waiting time and it will provide the chance of a shared evaluation by GPs and Neurologists.

No conflict of interest.

